# P-1594. Predictive models to improve antibiotic decision-making in the Emergency Department for Extended-Spectrum β-Lactamase-producing *Enterobacterales* infections

**DOI:** 10.1093/ofid/ofae631.1761

**Published:** 2025-01-29

**Authors:** Olga Kuzmich, Xihan Zhao, Jerald Cherian, Sara E Cosgrove, Jeremiah S Hinson, Eili Klein

**Affiliations:** Johns Hopkins, Baltimore, Maryland; Johns Hopkins University, Baltimore, Maryland; Johns Hopkins University School of Medicine, Baltimore, Maryland; Johns Hopkins School of Medicine, Baltimore, MD; Johns Hopkins University School of Medicine, Baltimore, Maryland; Johns Hopkins School of Medicine, Baltimore, MD

## Abstract

**Background:**

Extended-Spectrum β-Lactamase-producing Enterobacterales (ESBL-E) infections are associated with high morbidity and mortality, often due to delayed initiation of carbapenem therapy. Carbapenems must be used judiciously to prevent emergence of resistant organisms. We developed a machine learning algorithm (MLA) to identify patients at increased risk for ESBL-E infections in the emergency department (ED).

**Figure 1:**
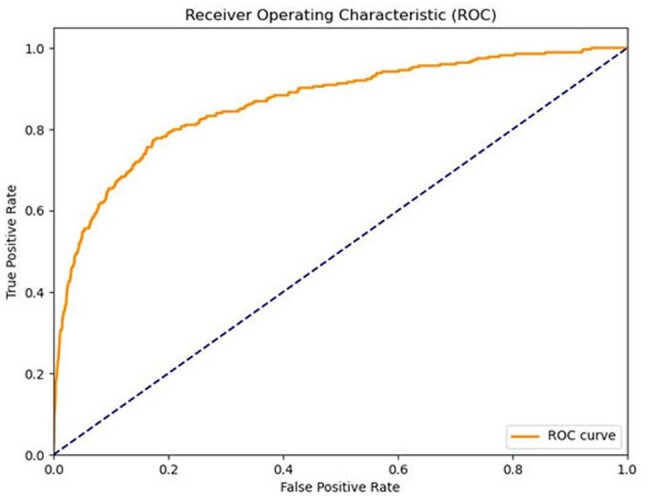
Receiver Operating Characteristic (ROC) curve for predicting Extended-Spectrum β-Lactamase-producing Enterobacterales (ESBL-E)

Receiver Operating Characteristic (ROC) curve for predicting Extended-Spectrum β-Lactamase-producing Enterobacterales (ESBL-E) infections in Emergency Department Patients using a gradient boosting machine learning model. The area under the curve (AUC) is 0.8, indicating good discrimination between patients with and without ESBL-E. Higher True Positive Rate (TPR) at a low False Positive Rate (FPR) signifies better model performance.

**Methods:**

Adults (≥18) who visited an ED in the Johns Hopkins Health System between 1/2019 and 4/2024 and received Gram-negative therapies appropriate for sepsis or had documented ESBL-E infections were included. Timestamped ED vital signs, healthcare utilization, healthcare worker (HCW)-mediated patient connections, medication history, comorbidities and laboratory results populated prior to first antibiotic order were used as predictors; our primary outcome was ESBL-E infection defined by positive culture. The probability of ESBL-E infection was estimated using a gradient boosting machine learning algorithm (MLA). Because of the time-dependent nature of increasing ESBL-E infections, the MLA was derived using data from encounters prior to 7/1/2023 and evaluated in the remaining encounters using area under the operating characteristics curve (AUC) analysis.

**Table 1:**
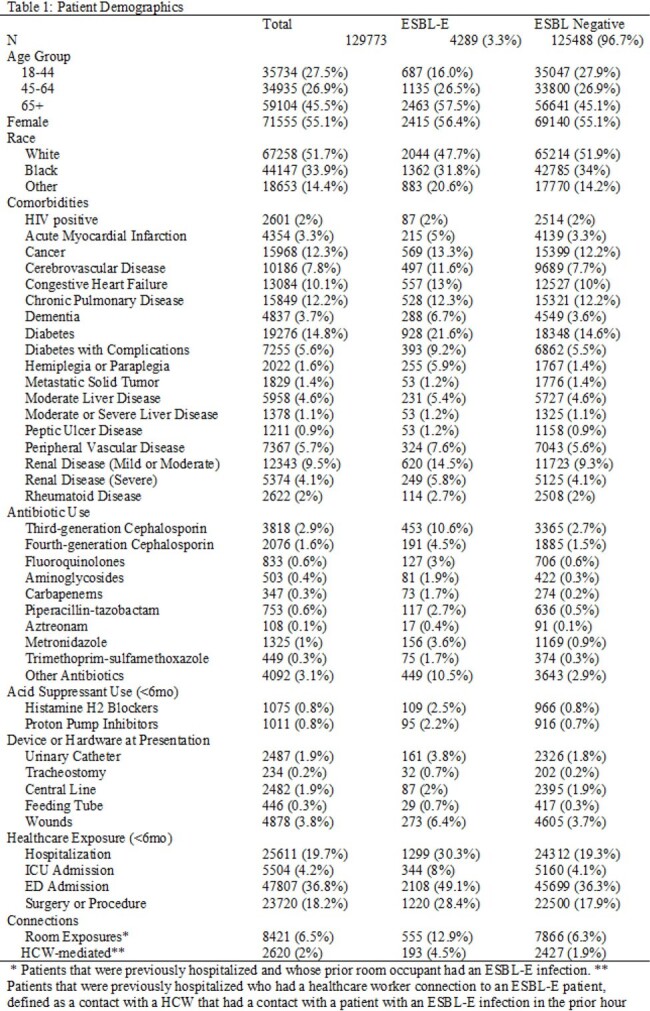
Patient Cohort Demographics

**Results:**

In total there were 129,773 patient encounters included, of which 4289 (3.3%) had a positive ESBL-E sample collected during their ED encounter. Out-of-sample predictive accuracy was high (AUC 0.80; Figure). Prior HCW-mediated connections to ESBL-positive patients and prior exposure (≤6 months) to carbapenems, cephalosporins and proton pump inhibitors were the strongest predictors of ESBL-E infection. Abnormal vital signs, increased age, and prior exposure to any antibiotic (prior 6 months) were also important factors in prediction accuracy.

**Conclusion:**

An MLA using data readily available from the EHR at the time of antibiotic ordering provided good discrimination between ED patients with and without ESBL-E infection. Further refinement and implementation of MLA-driven decision-support in the EDs could drive more targeted use of broad-spectrum antibiotics and improved patient outcomes.

**Disclosures:**

**Jeremiah S. Hinson, MD, PhD**, Beckman Coulter: Advisor/Consultant|Beckman Coulter: Grant/Research Support|Beckman Coulter: Stocks/Bonds (Private Company)

